# Risk factors for severe postpartum hemorrhage: a case-control study

**DOI:** 10.1186/s12884-016-1217-0

**Published:** 2017-01-10

**Authors:** Lill Trine Nyfløt, Irene Sandven, Babill Stray-Pedersen, Silje Pettersen, Iqbal Al-Zirqi, Margit Rosenberg, Anne Flem Jacobsen, Siri Vangen

**Affiliations:** 1 Division of Gynecology and Obstetrics, Oslo University Hospital, Rikshospitalet, P.O.box 4950, Nydalen, 0424 Oslo Norway; 2 Institute of Clinical Medicine, Faculty of Medicine, University of Oslo, P.O. box 1171, Blindern, 0318 Oslo Norway; 3 Department of Gynecology and Obstetrics, Drammen Hospital, P.O.box 800, 3004 Drammen, Norway; 4 Oslo Centre of Biostatistics and Epidemiology, Research Support Services, Sogn Arena, P.O.box 4950, Nydalen, 0424 Oslo Norway; 5 Norwegian National Advisory Unit on Women’s Health, Oslo University Hospital, P.o.box 4950, Nydalen, 0424 Oslo Norway

**Keywords:** Postpartum hemorrhage, Case-control study, Predictors, Risk factors, Obstetric interventions, High-risk, Prediction, Prevention

## Abstract

**Background:**

In high-income countries, the incidence of severe postpartum hemorrhage (PPH) has increased. This has important public health relevance because severe PPH is a leading cause of major maternal morbidity. However, few studies have identified risk factors for severe PPH within a contemporary obstetric cohort.

**Methods:**

We performed a case-control study to identify risk factors for severe PPH among a cohort of women who delivered at one of three hospitals in Norway between 2008 and 2011. A case (severe PPH) was classified by an estimated blood loss ≥1500 mL or the need for blood transfusion for excessive postpartum bleeding. Using logistic regression, we applied a pragmatic strategy to identify independent risk factors for severe PPH.

**Results:**

Among a total of 43,105 deliveries occurring between 2008 and 2011, we identified 1064 cases and 2059 random controls. The frequency of severe PPH was 2.5% (95% confidence interval (CI): 2.32–2.62). The most common etiologies for severe PPH were uterine atony (60%) and placental complications (36%). The strongest risk factors were a history of severe PPH (adjusted OR (aOR) = 8.97, 95% CI: 5.25–15.33), anticoagulant medication (aOR = 4.79, 95% CI: 2.72–8.41), anemia at booking (aOR = 4.27, 95% CI: 2.79–6.54), severe pre-eclampsia or HELLP syndrome (aOR = 3.03, 95% CI: 1.74–5.27), uterine fibromas (aOR = 2.71, 95% CI: 1.69–4.35), multiple pregnancy (aOR = 2.11, 95% CI: 1.39–3.22) and assisted reproductive technologies (aOR = 1.88, 95% CI: 1.33–2.65).

**Conclusions:**

Based on our findings, women with a history of severe PPH are at highest risk of severe PPH. As well as other established clinical risk factors for PPH, a history of severe PPH should be included as a risk factor in the development and validation of prediction models for PPH.

## Background

Severe postpartum hemorrhage (PPH) is the largest contributor to maternal morbidity worldwide, accounting for 50–75% of all such cases [1–4]. Consequently, PPH has received increasing attention as a quality indicator for obstetric care. Furthermore, evidence exists that the incidence of PPH is increasing in high-income countries [5–12]. An increase in the prevalence of known maternal and obstetric risk factors for PPH could play a role, but the supporting evidence from the published studies is limited. For example, in a Canadian study [7], induction of labor, augmentation of labor, and cesarean section partially explained the increasing rate of PPH. These findings may indicate that women undergoing these interventions need closer monitoring for severe PPH in the early postpartum period.

Several risk factors for PPH are known, such as multiple pregnancy, operative delivery and chorionamnionitis, however PPH may occur among patients with no known risk factors [13, 14]. Our ability to reduce the risk of PPH depends on ongoing investigations of previously unaccounted for causes and risk factors.

The primary aim of the study was to evaluate risk factors for severe PPH, taking into consideration pre-pregnancy, antenatal and intrapartum variables.

## Methods

The source population was defined as pregnant women living in the metropolitan area of the Oslo and Buskerud municipality who were admitted to two university hospitals in Oslo (Ullevaal or Rikshospitalet) or Drammen Hospital for delivery between January 1, 2008 and December 31, 2011. From this source population, we performed a retrospective case-control study. We identified 1064 cases of severe PPH through birth suite records and hospital databases. Severe PPH was defined as blood loss ≥1500 mL or the need for blood transfusion for excessive bleeding at the time of delivery. Blood transfusion for excessive bleeding was defined as a blood transfusion given for a likely PPH ≥1500 mL due to clinical symptoms and signs of anemia or hemodynamic decompensation after delivery. We excluded women who received a blood transfusion because of postpartum anemia, without evidence of excessive hemorrhage. The attending physician or midwife estimated the blood loss visually in all three hospitals. Controls were a random sample of all deliveries without severe PPH from the same source population and period of time as the cases, comprising a total of 2059 deliveries. We selected random controls after removing the cases of severe PPH from the total number of deliveries at the three hospitals. Weighting was done according to the total number of deliveries in each hospital during the study period, resulting in control fractions from Rikshospitalet, Ullevaal, and Drammen of 21%, 62%, and 17%, respectively. Considering two controls per case, we estimated the number of controls needed from each hospital according to the number of delivering women in each hospital compared to the total number. This rendered sampling fractions at Rikshospitalet, Ullevaal, and Drammen of 4.8%, 5.2%, and 4.7%, respectively. The random sample was generated in STATA version 11.0 (Stata Corp LP, College Station, TX, USA).

Registration of patient data was based on information from 1) the hospitals’ medical records; 2) maternity databases (Obstetrix® from Siemens AG, Oslo, Norway and Partus® from Clinsoft, Oslo, Norway), and 3) birth suite records containing labor and delivery outcomes on all deliveries, including the volume of blood loss during delivery. If a woman had more than one delivery, the second and subsequent pregnancies were excluded to limit repeated correlated measurements.

In this study, we distinguished between causes of and risk factors for PPH; no direct causes were included in the risk factor analyses. Causes of severe PPH were classified as Tone (uterine atony, uterine inversion and abruption of the placenta), Tissue (retained placenta and retained parts of placenta, and abnormal placentation), Trauma (uterine rupture, birth canal trauma, and surgical trauma), and Thrombin (coagulation disorders). We registered up to two causes for each case if both were considered to be main causes, except in cases labeled as atony due to retained placenta which were reported as a retained placenta. A retained placenta necessitating a manual or operative delivery of the placenta was classified as a retained placenta. Cases with retained placental tissues diagnosed in the operating theatre or by ultrasound and needing surgical or manual removal, were classified as retained placental tissue. Abnormal placentation was defined as placenta accreta, increta or percreta. We identified cases caused by abnormal placentation post-delivery by reviewing medical records and pathology reports.

Based on literature review, we selected potential risk factors for consideration in our analyses. Pre-pregnancy factors included marital status, ethnicity, uterine anomalies (septated uterus, uni- or bicornuate uterus, uterus didelphys), previous uterine surgery (myomectomy and septal removals), previous cesarean section, previous severe PPH (≥1500 mL), and uterine fibromas. Current pregnancy conditions included maternal age, ethnicity (country of origin), pre-pregnancy body mass index (BMI), anemia in start of pregnancy (hemoglobin ≤9 g/dL), assisted reproductive technology (in vitro fertilization [IVF] or intra-cytoplasmic sperm injection [ICSI]), multiple pregnancy, gestational diabetes (insulin treated or diet regulated), use of anticoagulant medications such as low molecular weight heparin (LMWH) in pregnancy, polyhydramnios, severe preeclampsia or HELLP-syndrome, and premature rupture of membranes (PROM). Intrapartum factors included maternal fever (>38 °C) during delivery, mode of delivery, induction of labor, labor augmentation with oxytocin, and infant birth weight. Maternal age, BMI, and infant birth weight were considered as continuous variables for inclusion in the final model and categorical variables for descriptive purposes. Age was divided into 5-year groups, using 20–24 years as the reference group. BMI was categorized using the World Health Organization (WHO)’s classification, with a BMI of 18.5-24.9 kg/m^2^ as the reference. Infant birth weight was dichotomized into ≥4500 g or <4500 g, according to the definition of fetal macrosomia [15]. Furthermore, we used WHO’s world regions to classify ethnic origin [16].

The data were entered into a database built in EpiData Version 3.1. (EpiData Association, Odense, Denmark). The medical records were re-examined if we observed data outliers or categorization errors for independent and dependent variables. All medical records were reviewed by two investigators (LTN and SP).

For our sample size estimation, we considered maternal age and induction of labor as potential risk factors for severe PPH. For both sample size estimates, we considered a type 1 error of 5%, a power of 80%, and a case:control ratio of 1:2. Regarding maternal age, in Oslo, approximately 18% of parturients are 35 years or older [17]. We hypothesized that women in this age group had a 1.4-fold increased risk of severe PPH compared to younger women [1, 18]. With a minimum detectable odds ratio (OR) = 1.4, we would need at least 656 cases and 1312 controls for a total of 1968 patients. Regarding induction of labor, in Norway, the frequency of labors induced with oxytocin is 3.4% according to figures from The Medical Birth Registry of Norway [17]. In a population-based study from Norway examining risk factors for severe PPH, the total rate of induction of labor was 10.8% [18]. We estimated that the frequency of induction of labor would be around the mean of these rates. Assuming a 1.6-fold increased risk of severe PPH in induced labor [1, 18], we would need at least 698 cases and 1396 controls, totally 2094 patients, when considering a minimum detectable OR = 1.6. Thus, for this case–control study, we decided to include at least 2400 deliveries (800 cases and 1600 controls).

We analyzed the data according to a pragmatic strategy [19], which means priority was not given to a specific etiological hypothesis. Univariable analysis was done to assess candidate variables as risk factors for severe PPH, and the associations between potential risk factors and severe PPH was quantified by the OR and 95% confidence interval (CI). The multivariable analysis was preceded by estimation of collinearity between risk factors. When collinearity existed between two variables, we omitted the one with least clinical relevance. Finally, multivariable logistic regression with manual backward elimination was used to identify independent risk factors for severe PPH [20]. Our criteria for sequential elimination of candidate risk factors were the variables’ strength and significance on the association with severe PPH, and optimal calibration and discrimination of the model. The continuous variables of maternal age, BMI, and birth weight were introduced into the model in its logged form as they were linearly associated with the outcome. However, birth weight is presented dichotomized in Table 3 as this was judged clinically more interesting.

Because we were applying a pragmatic strategy, the predictive accuracy of the model was evaluated by *calibration* and *discrimination* [21]. *Calibration*, which measures the ability of the model to assign the appropriate risk, was evaluated by the Hosmer and Lemeshow (H-L) goodness-of-fit test. A statistically non-significant H-L result (*P* > 0.05) suggests that the model predicts accurately on average. *Discrimination*, which measures the model’s ability to differentiate between individuals with and without severe PPH, was evaluated by analysis of the area under the receiver operating characteristic (ROC) curve. If the area under the curve is greater than 0.7, it can be concluded that the model has an acceptable discriminatory capability. All statistical analysis was performed using STATA version 11.0 (Stata Corp LP, College Station, TX, USA). We used Strengthening the Reporting of Observational Studies in Epidemiology (STROBE) guidelines in reporting our case–control study [22].

## Results

From the source population of 43,105 deliveries, we identified 1064 women with a recorded PPH of ≥1500 mL or blood transfusion, giving a frequency of 2.5% (95% CI: 2.32–2.62). The identified causes of severe PPH are listed in Table 1. The most common cause was uterine atony (60.4%), while we identified retained placenta in 19.8% of the cases. Abnormal placentation was diagnosed post-delivery in 4.4% of the cases.

**Table 1:** Causes of severe postpartum hemorrhage (*N* = 1064)

Cause^a^	
Tone	671 (63.0%)
Uterine atony^b^ Uterine inversion Abruption of placenta	643 (60.4%) 5 (0.5%) 23 (2.2%)
Tissue	380 (35.7%)
Retained placenta Retained placental tissue Abnormal placentation (accreta, increta, percreta)	211 (19.8%) 122 (11.4%) 47 (4.4%)
Trauma	189 (17.8%)
Birth canal trauma	114 (10.7%)
Surgical trauma during caesarean delivery	63 (5.9%)
Uterine rupture	12 (1.1%)
Thrombin	16 (1.5%)
Disseminated intravascular coagulation	8 (0.8%)
Pre-existing coagulation disorders	8 (0.8%)

Data presented as n (%)

^a^23% of the cases had two major causes listed

^b^excluding cases with atony due to retained placental tissue

The study population comprised a total of 1064 cases of severe PPH and 2059 random controls without severe PPH. The distribution of potential risk factors is presented in Table 2. Europe, the United States, and Oceania were countries of origin for the majority of cases and controls (78.8% vs. 81.7%, respectively). The median (interquartile ranges) values for maternal age, BMI, and birthweight were similar among cases and controls; maternal age: 32 (29–36) years vs. 32 (29–35) years respectively; pre-pregnancy BMI: 23.1 (21.0–26.1) kg/m^2^ vs. 22.8 (20.8–25.7) kg/m^2^ respectively; and birthweight: 3546 (3075–3930) g vs. 3465 (3120–3834) g respectively. In the univariable analysis, severe PPH was more likely among women with the following medical and obstetric characteristics: primiparity, women who were married or cohabiting, previous severe PPH, previous uterine surgery, known uterine anomaly, multiple gestation, IVF/ICSI pregnancies, anemia, gestational diabetes mellitus, uterine fibroma, polyhydramnios, receipt of anticoagulant medication, and severe pre-eclampsia or HELLP syndrome. Severe PPH was more likely among women with these intrapartum and delivery characteristics: instrumental vaginal delivery, in-labor cesarean delivery, induced labor, labor augmentation with oxytocin, fever during labor, PROM, and infant birth weight ≥4500 g.

**Table 2:** Clinical profile of women with severe postpartum hemorrhage versus controls

	Severe PPH (*N* = 1064)	Controls (*N* = 2059)	OR	95% CI	*P* -value
Age (years)					
14 – 19	12 (1.1%)	13 (0.6%)	2.23	0.96 – 5.17	0.061
20 – 24	60 (5.6%)	145 (7.0%)	Ref.		
25 –29	240 (22.6%)	507 (24.6%)	1.14	0.82 – 1.60	0.435
30 –34	395 (37.1%)	770 (37.4%)	1.24	0.90 –1.71	0.194
35–39	283 (26.6%)	501 (24.3%)	1.36	0.98–1.91	0.068
≥40	74 (7.0%)	123 (6.0%)	1.45	0.96–2.21	0.078
Parity					
0	622 (58.5%)	1007 (48.9%)	1.54	1.30 – 1.82	<0.001
1	296 (27.8%)	738 (35.8%)	Ref.		
2	96 (9.0%)	229 (11.1%)	1.04	0.79 – 1.37	0.752
≥3	50 (4.6%)	85 (4.1%)	1.47	1.01 – 2.13	0.045
Ethnicity					
Europe/USA/Oceania	838 (78.8%)	1682 (81.7%)	Ref.		
Middle-East/North-Africa	50 (4.6%)	122 (5.9%)	0.82	0.58 – 1.15	0.259
Latin-America	14 (1.3%)	22 (1.1%)	1.28	0.65 – 2.51	0.477
Asia	99 (9.3%)	151 (7.3%)	1.31	1.01 – 1.72	0.044
Sub-Saharan Africa	63 (5.9%)	82 (4.0%)	1.54	1.10 – 2.16	0.012
BMI (kg/m^2^)					
<18.5	42 (4.0%)	80 (3.9%)	1.08	0.74 – 1.59	0.688
18.5 – 24.9	639 (60.1%)	1317 (64.0%)	Ref.		
25.0 – 29.9	205 (19.3%)	378 (18.4%)	1.12	0.92 – 1.36	0.262
30.0 – 34.9	82 (7.7%)	145 (7.0%)	1.16	0.87 – 1.55	0.295
35.0 – 39.9	25 (2.4%)	36 (1.8%)	1.43	0.85 – 2.40	0.176
≥40	7 (0.7%)	14 (0.7%)	1.03	0.41 – 2.56	0.949
Married/cohabitant	973 (91.4%)	1932 (94.1%)	0.70	0.53 – 0.93	0.014
Pre-pregnancy conditions					
Uterine anomaly	16 (1.5%)	13 (0.6%)	2.40	1.15 – 5.01	0.020
Uterine surgery	19 (1.8%)	11 (0.5%)	3.38	1.60 – 7.14	0.001
Previous cesarean	126 (11.8%)	221 (10.7%)	1.12	0.88 – 1.41	0.350
Previous severe PPH	66 (6.2%)	21 (1.0%)	6.42	3.90 – 10.6	<0.001
Obstetric factors					
Multiple pregnancy	94 (8.8%)	52 (2.5%)	3.74	2.64 – 5.29	<0.001
IVF/ICSI	115 (10.8%)	82 (4.0%)	2.92	2.18 – 3.92	<0.001
Anemia (Hb ≤ 9.0 g/dL)	74 (7.0%)	38 (1.9%)	4.11	2.76 – 6.13	<0.001
Gestational diabetes mellitus	46 (9.4%)	58 (2.8%)	1.56	1.05 – 2.31	0.027
Uterine fibroma	52 (4.9%)	38 (1.9%)	2.73	1.79 – 4.18	<0.001
Polyhydramnios	16 (1.5%)	12 (0.6%)	2.60	1.23 – 5.52	0.013
Anticoagulant medication	51 (4.8%)	22 (2.1%)	4.66	2.81 – 7.73	<0.001
Severe pre-eclampsia or HELLP syndrome	50 (4.7%)	28 (2.6%)	3.58	2.24 – 5.71	<0.001
Intrapartum factors					
Mode of delivery					
Spontaneous vaginal	507 (47.6%)	1358 (66.0%)	Ref.		
Instrumental vaginal	212 (20.0%)	25 (12.2%)	2.26	1.83 – 2.79	<0.001
In-labor cesarean	248 (23.3%)	245 (11.9%)	2.71	2.21 – 3.32	<0.001
Elective cesarean	97 (8.6%)	205 (10.0%)	1.27	0.97 – 1.65	0.077
PROM	127 (12.0%)	169 (8.2%)	1.51	1.19 – 1.93	0.001
Fever (temp > 38 °C) in labor	75 (7.1%)	60 (2.9%)	2.53	1.78 – 3.58	<0.001
Labor augmentation	587 (55.2%)	797 (38.7%)	1.95	1.68 – 2.26	<0.001
Labor induction	349 (32.8%)	402 (19.5%)	2.01	1.70 – 2.38	<0.001
Birth weight > 4500 g	51 (4.8%)	57 (2.8%)	1.77	1.20 – 2.60	0.004

Data presented as *n* (%), odds ratio (OR) and 95% confidence intervals (CI)

*BMI* body mass index, *Hb* hemoglobin, *IVF/ICSI* in vitro fertilization/intra-cytoplasmic sperm injection, *HELLP* hemolysis, elevated liver enzymes, low platelet count, *PROM* premature rupture of membranes, *PPH* postpartum hemorrhage

Risk factors independently associated with severe PPH are presented in Table 3. The strongest independent risk factors were a history of severe PPH (adjusted odds ratio (aOR) = 8.97; 95% CI: 5.25–15.33), anticoagulant drugs in pregnancy (aOR = 4.78; 95% CI: 2.72–8.41), anemia diagnosed in the start of pregnancy (aOR = 4.27; 95% CI: 2.79–6.54), severe pre-eclampsia or HELLP syndrome (aOR = 3.03; 95% CI: 1.74–5.27), uterine fibromas (OR = 2.71; 95% CI: 1.69–4.35), and multiple pregnancy (aOR = 2.11; 95% CI: 1.39–3.22). The H-L goodness-of-fit test was non-significant (chi-square = 12.99, *P* = 0.1122), indicating satisfactory model fit. The area under the ROC curve was 0.7173, indicating an acceptable discriminatory capability.

**Table 3:** Multivariable logistic model for severe postpartum hemorrhage

Independent risk factors	Adjusted OR	95% CI	*P* – value
Previous severe PPH	8.97	5.25 – 15.33	<0.001
Anticoagulant medication	4.79	2.72 – 8.41	<0.001
Anemia (Hb ≤ 9.0 g/dL)	4.27	2.79 – 6.54	<0.001
Severe preeclampsia or HELLP syndrome	3.03	1.74 – 5.27	<0.001
Uterine fibromas	2.71	1.69 – 4.35	<0.001
Multiple pregnancy	2.11	1.39 – 3.22	<0.001
Mode of delivery			
Spontaneous vaginal	Ref.		
Instrumental vaginal	1.50	1.17 – 1.93	0.001
In-labor cesarean	1.95	1.53 – 2.47	<0.001
Elective cesarean	1.66	1.22 – 2.24	0.006
IVF/ICSI	1.88	1.33 – 2.65	<0.001
Fever (>38 °C)	1.88	1.28-2.75	0.001
Labor induction	1.69	1.39 – 2.05	<0.001
Labor augmentation	1.59	1.32 – 1.91	<0.001
Birth weight > 4500 g	1.46	1.01 – 2.12	0.046
Primiparity	1.20	0.99 – 1.44	0.055

Area under ROC curve = 0.7173, Hosmer-Lemeshow *χ* ^2^ = 12.99, *p* = 0.1122

*PPH* postpartum hemorrhage, *Hb* hemoglobin, *HELLP* hemolysis, elevated liver enzymes, low platelet count, *IVF/ICSI* in vitro fertilization/intra-cytoplasmic sperm injection

Finally, we performed an additional sensitivity analysis where the outcome was limited to an estimated postpartum blood loss ≥1500 mL. When considering the same risk factors, the results of the sub-analysis did not change our conclusions (Appendix: Table 4). Goodness-of-fit of the model was indicated by a non-significant H-L test (*χ* ^2^ = 8.77, p = 0.3618), and a borderline discriminatory capability (area under the ROC curve = 0.6927).

## Discussion

In this case-control study, we evaluated risk factors for severe PPH. A history of severe PPH was the strongest independent risk factor in our study. Our findings suggest that women with increased risk of severe PPH can be identified when antepartum and intrapartum variables are considered. Furthermore, retained placental tissue was a much more frequent cause of severe PPH than previously reported.

## Strengths and limitations

The main strength of this case-control study was the quality of the data source. By reviewing medical records, we were able to evaluate potential risk factors for severe PPH using a broad selection of clinical variables which may not be easily retrieved from registries. In addition, reading the medical records enabled us to present accurate information on the causes of severe PPH and especially on the rate of retained placental tissue (including abnormal placentation) in the cases. We lacked data on women with suspected abnormal placentation prior to delivery as there was little awareness around pre-delivery diagnosis of abnormal placentation in Norway during the study period. Abnormal placentation could therefore not be included as a risk factor for severe PPH in our model.

Assessing risk factors in retrospect is a limitation in this study. In order to minimize selection bias, we selected all cases of severe PPH and a random sample of controls from the same source population. There is a possibility that some cases were misclassified. Blood loss was estimated visually in the three hospitals included in our study, and the blood loss may have been underestimated [23, 24]. However, we had further data in medical records to assess the severity of the hemorrhage by need for blood transfusion. There is a possibility of information bias due to misclassification of the exposures. However, neither the resident obstetrician nor midwife were aware of our research questions, therefore the potential for misclassification of risk factors would be non-differential creating a bias toward the null [20]. The sample size provided adequate power to highlight risk factors mildly associated with severe PPH, such as infant birth weight. However, we did not account for multiple testing in the power analysis and the smallest detectable odds ratios may have been underestimated.

We based our results on deliveries in three hospitals in or close to Oslo, Norway, and our results may not necessarily be generalizable to other delivery populations in well-resourced countries. The majority of the women in this study had European, USA, or Oceanian ethnicity, and was non-obese and married. These characteristics reflect a society with a low proportion of women with low socioeconomic status and fewer immigrants compared to other well-resourced countries. Furthermore, the hospitals mainly reflect an urban setting, but urban-rural differences in Norway are small and home births are an exception.

### Important risk factors for severe PPH

The strongest risk factor in our study was a history of severe PPH. Women with a history of severe PPH had nine-fold increased odds of severe PPH in their index pregnancy. Previously reported estimates on recurrence risk are mainly from register-based studies [25–27], presenting lower estimates between 2.2 and 3.3. Having a history of PPH may be less well-reported to the registries than in our data sources. Likewise, a validation study from Australia [28] reported a recurrence rate of 28% from medical record audits, while the reported recurrence rate in register based data was 18%. The causes of PPH recurrence were investigated in a population study from Sweden [27]. They reported that recurrence risk could not be explained by known risk factors for PPH, suggesting that recurrence may depend on environmental and genetic factors. Another study by the same authors [29] reported that, among vaginal deliveries, 18% of the variation in PPH liability may be explained by maternal genetic factors. Alterations in maternal hemostasis and oxytocin signaling at the myometrial level were postulated as possible pathways for a maternal genetic predisposition to PPH.

A close to five-fold increased risk of severe PPH was found in women using anticoagulant drugs in pregnancy. The obstetric guidelines in Norway recommend stopping anticoagulants at the onset of labor or 12 h before a planned cesarean delivery. Our finding is in line those from a Swedish study [30]. In this study, the risk of PPH was increased three-fold for women using anticoagulants in pregnancy. Other studies, however, have not found any significantly increased risk of severe PPH associated with the use of anticoagulant drugs [31, 32]. Differences in anticoagulant drug regimens may explain why this association has not been consistently shown across these studies. Of note, in our analyses, we did not account for drug dosing or the time interval between last anticoagulant dose and PPH onset.

Obstetric interventions, such as augmentation and induction of labor, instrumental vaginal delivery, and cesarean delivery, were all significantly associated with severe PPH. A potential for risk reduction is likely if the use of these interventions are limited to situations where existing evidence supports their safe use. In line with previous studies, compared with spontaneous vaginal delivery, the odds of severe PPH were higher among women undergoing either in-labor or planned cesarean delivery [1, 33]. Previous studies have shown conflicting results [1, 34–36] on whether labor augmentation is a risk factor independent of induction. Oxytocin receptor desensitization may explain why labor augmentation with oxytocin is associated with uterine atony leading to PPH [37–39]. The association between oxytocin administration and PPH has been reported to be dose related and evidence-based guidelines are needed to determine optimal oxytocin regimens for labor augmentation to lessen the risk of atonic PPH [34].

We found that an IVF or ICSI pregnancy was an independent risk factor for severe PPH. This association has been poorly described, and previous studies observing an association between assisted reproductive technology and severe PPH are from retrospective infertility cohorts [40–42]. Events around the implantation of the placenta, such as low placement of the embryo into the uterus and endometrial function disturbances, could play a role here. As the use of assisted reproductive technology is increasing, more research to investigate the potential association between IVF and ICSI pregnancies and PPH is warranted.

### Frequency and causes of severe PPH

In our study, the frequency of severe PPH was 2.5%. A population-based study from Norway using the Norwegian Birth Registry [18], reported a severe PPH rate of 1.1%. The difference can be explained by a tendency to underreport cases of severe PPH to the birth registries. A validation study from Australia [43] showed an underreporting of obstetric hemorrhage with a sensitivity of only 74% in population-based registry studies.

In a study examining risk factors and outcomes of massive blood transfusions during delivery [44], abnormal placentation was reported to be the most common cause (26.6%). The same study reported that a disproportionate number of women who received a massive blood transfusion experienced severe maternal morbidity, illustrating why abnormal placentation is becoming a major concern in obstetrics. In our study, we identified placental problems (retained placenta, retained placental tissue and abnormal placentation) as the cause of severe PPH in nearly 36% of cases. Abnormal placentation was diagnosed post-delivery in 4.4% of cases. In addition, abnormal placentation may have been present for some cases diagnosed as retained placenta as pathology reports are not available for the majority of these cases. Retained placental tissue, including abnormal placentation, has been estimated to cause approximately 10% of all PPHs [8, 24]. Our findings suggest that retained placental tissue may be a more prominent cause of severe PPH than previously reported. False reporting of cases caused by retained placental tissue as atonic bleeding in registries could explain this discrepancy. In a validation study examining the contribution of uterine atony to PPH [28], the incidence of uterine atony was overestimated by 10% when registry data were compared with clinical data. Access to detailed medical information enabled us to accurately estimate the proportion of cases with placental problems in our population. We believe the high rate of placental problems revealed in the current study could reflect an increasing rate of placental problems.

## Conclusion

In this study, the strongest risk factors for severe PPH was a history of severe PPH, anticoagulant medication, anemia, severe preeclampsia or HELLP syndrome, uterine fibromas, and multiple pregnancy. Including these risk factors in clinical guidelines could help to identify women with high risk of severe PPH prior to delivery. By identifying these women, adequate resources and staff could be mobilized in preparation for severe bleeding at the time of delivery. Several of the identified risk factors in our study were related to medical and obstetric interventions such as anticoagulant medication, assisted reproductive technologies, labor induction, and labor augmentation with oxytocin. Further studies are needed to better understand the risk-benefit profile of each intervention on maternal outcomes. Risk factors identified in our study could be considered in future studies examining risk prediction models for severe PPH.

## Appendix

**Table 4:** Sensitivity analysis of women with an estimated postpartum hemorrhage ≥1500 mL

Independent risk factors	Adjusted OR	95% CI	*P* – value
Previous severe PPH	10.50	6.09 – 18.10	<0.001
Anticoagulant medication	4.33	2.39 – 7.88	<0.001
Anemia (Hb ≤ 9.0 g/dL)	2.43	1.45 – 4.08	0.001
Severe preeclampsia/HELLP	1.82	0.9 – 53.50	0.089
Uterine fibromas	2.66	1.58 – 4.46	<0.001
Multiple pregnancy	1.49	0.96 – 2.32	0.077
Mode of delivery			
Spontaneous vaginal	Ref		
Instrumental vaginal	1.10	0.82 – 1.47	0.409
In-labor cesarean	1.36	1.03 – 1.79	0.028
Elective cesarean	1.34	0.94 – 1.89	0.103
IVF/ICSI	1.78	1.21 – 2.61	0.004
Fever (>38 °C)	1.96	1.29 – 2.97	0.002
Labor induction	1.69	1.36 – 2.10	<0.001
Labor augmentation	1.65	1.32 – 2.05	<0.001
Birth weight > 4500 g	1.62	1.00 – 2.60	0.046
Primiparous	1.14	0.93 – 1.40	0.209

Area under ROC curve = 0.6927, Hosmer-Lemeshow *χ* ^2^ = 8.77, p = 0.3618

*PPH* postpartum hemorrhage, *Hb* hemoglobin, *HELLP* hemolysis, elevated liver enzymes, low platelet count, *IVF/ICSI* in vitro fertilization/intra-cytoplasmic sperm injection

## Abbreviations

BMI: Body mass index
CI: Confidence interval
HB: Hemoglobin
HELLP: Hemolysis, elevated liver enzymes, low platelet count
ICSI: Intra-cytoplasmic sperm injection
IVF: In vitro fertilization
LMWH: Low-molecular-weight-heparin
OR: Odds ratio
PPH: Postpartum hemorrhage
PROM: Prelabor rupture of membranes
SPPH: Severe postpartum hemorrhage
WHO: World Health Organization
